# Supplementary material for: Genome-Wide Evolutionary Characterization and Expression Analysis of Major Latex Protein (MLP) Family Genes in Tomato
Source: Int J Mol Sci. 2023 Oct 9;24(19):15005. doi: 10.3390/ijms241915005 (PMC10573222; doi:10.3390/ijms241915005)
Supplement: Supplementary file 1 [file ijms-24-15005-s001.zip › Supplementary data 1.pdf]

Title:Genome-wide evolutionary characterization and expression analysis of major latex protein (MLP) family genes in *tomato*

Supplementary Figures

Figure S1. Protein domain structures of SlMLPs.

Figure S2. Classification and number of the *cis*-elements in *SlMLP* promoters.

Figure S3. Expression analyses of *SlMLP* genes that are not associated with different abiotic stresses.

Figure S4. Venn diagram of *SlMLP* genes involved in cold, heat, salt and drought stresses.

Figure S5. Expression profiles of tomato SlMLP gene under various abiotic stress treatments, including high and low temperature.

Figure S6. Expression profiles of tomato SlMLP gene under various abiotic stress treatments, including salt and drought.

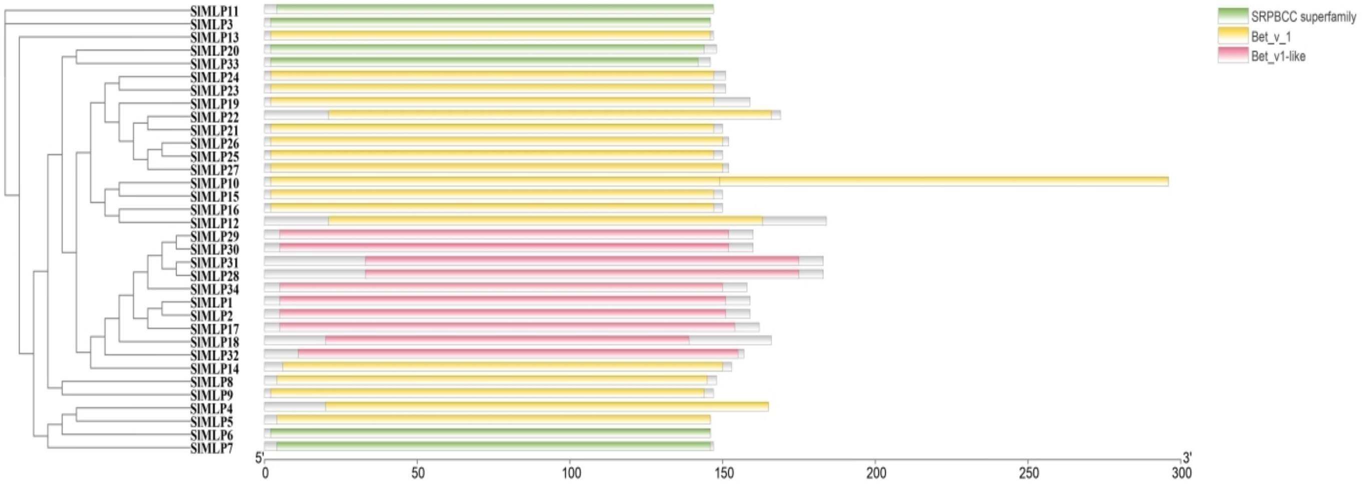

Figure S1. Protein domain structures of SlMLPs. The three conserved domains are marked in different colors.

The scale bars are shown at the bottom.

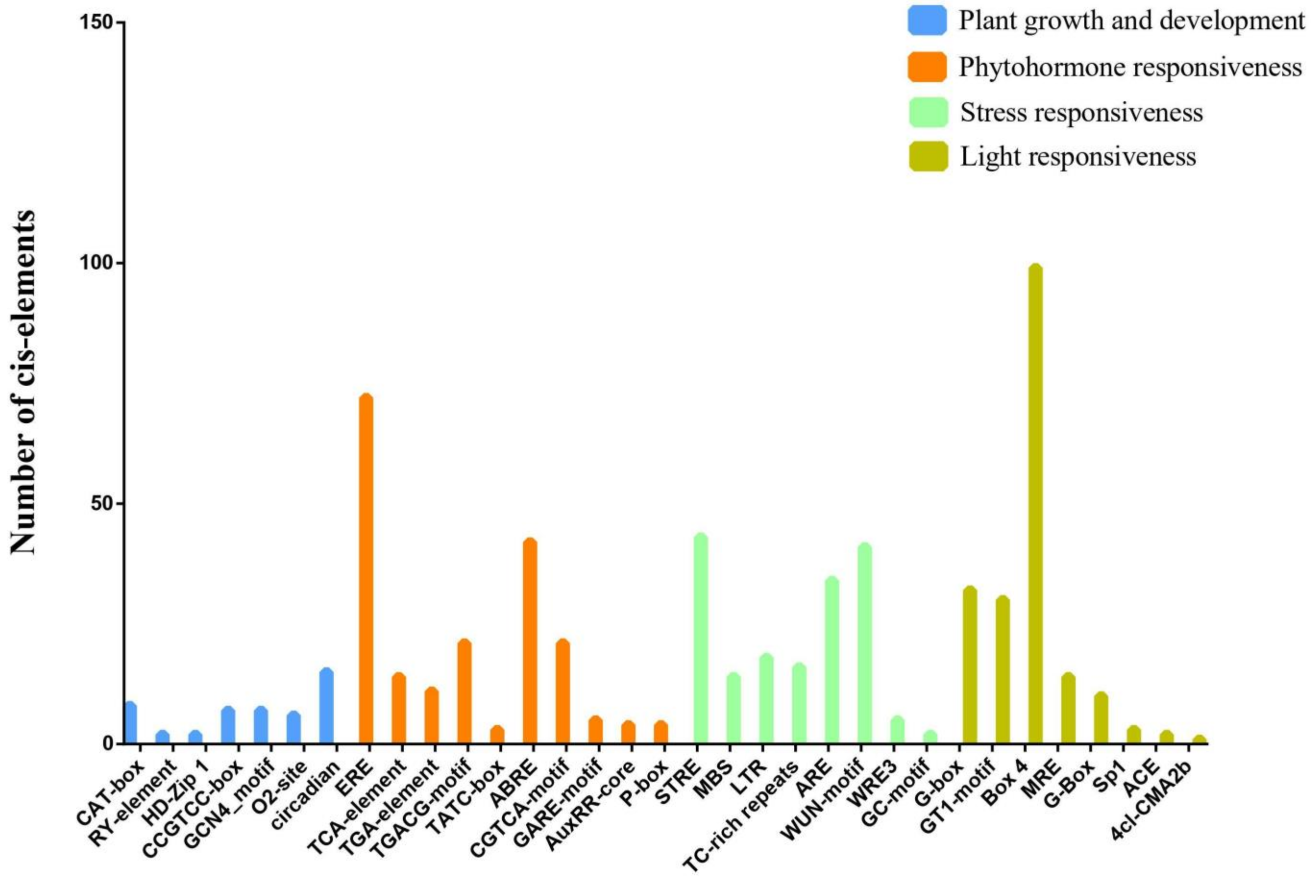

Figure S2. Classification and number of the *cis*-elements in *SlMLP* promoters.

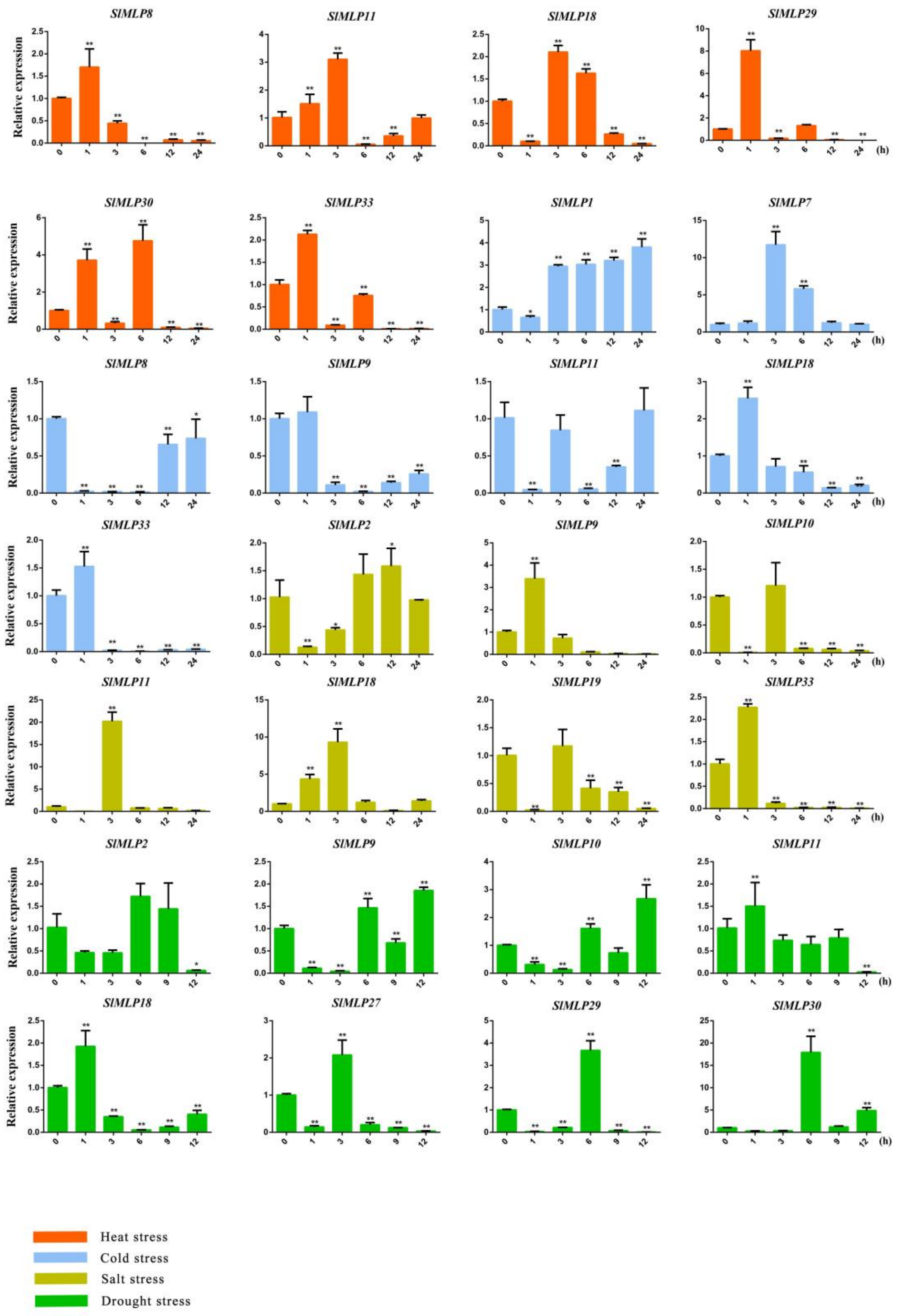

Figure S3. Expression analyses of *SlMLP* genes that are not associated with different abiotic stresses. *SlMLP* expressions under heat, cold, salt and drought stresses are indicated by orange, blue, brown and green colors, respectively. Value are the mean ± SD of three independent replicates. Asterisks represent significant differences between stress treatment (different time points) and Control (0 h) by Student's *t*-tests (* $P < 0.05$, ** $P < 0.01$).

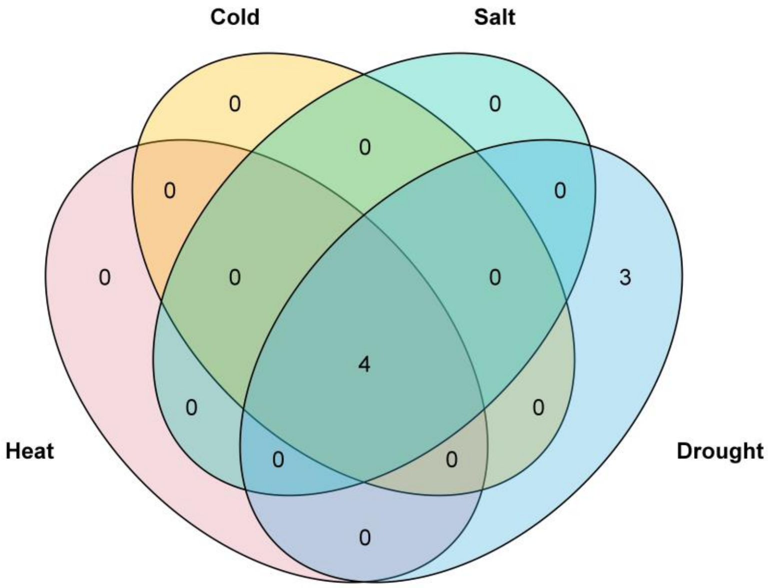

Figure S4. Venn diagram of *SlMLP* genes involved in cold, heat, salt and drought stresses.

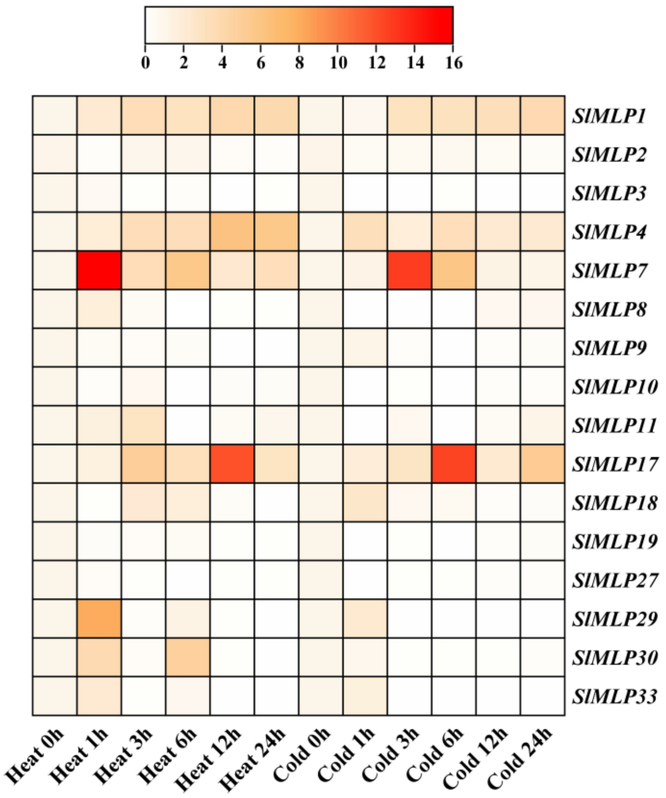

Figure S5. Expression profiles of tomato *SlMLP* gene under various abiotic stress treatments, including high and low temperature. Heat 0 h (1 h/3 h/6 h/12h/24h): heat treatment at 42 °C for 0 h (1 h/3 h/6 h/12h/24h); Cold 0 h (1 h/3 h/6 h/12 h/24 h): plants were exposed to 4 °C for 0 h (1 h/3 h/6 h/12 h/24h).

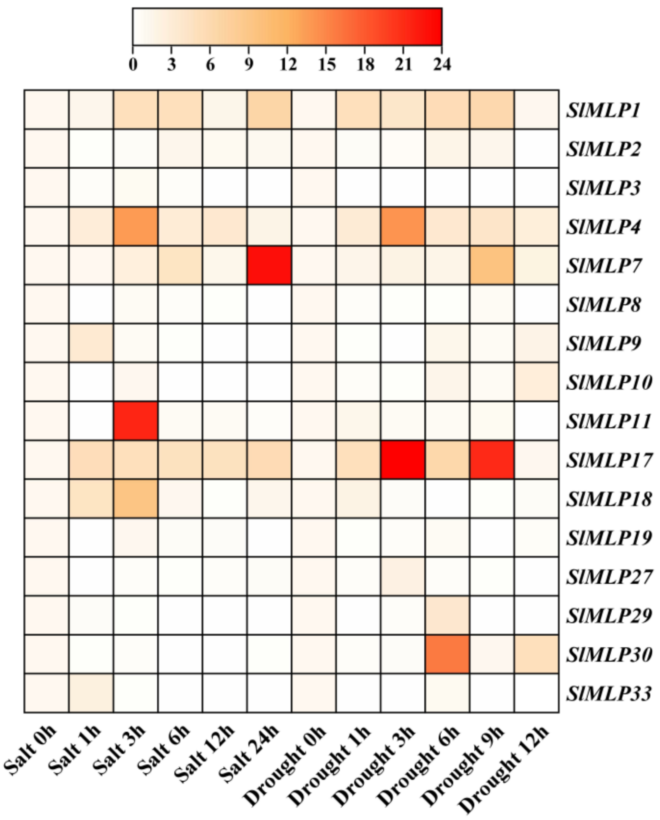

Figure S6. Expression profiles of tomato *SlMLP* gene under various abiotic stress treatments, including salt and drought. Salt 0 h (1 h/3 h/6 h/12h/24h): plants were watered with 200 mM NaCl for 0 h (1 h/3 h/6 h/12h/24h); Drought 0 h (1 h/3 h/6 h/9 h/12 h): plants were watered with 20% polyethylene glycol 6000 (polyethylene glycol) for 0 h (1 h/3 h/6 h/9 h/12 h).
