# Supplementary figures and images for: Genome-Wide Evolutionary Characterization and Expression Analysis of Major Latex Protein (MLP) Family Genes in Tomato
Source: Int J Mol Sci. 2023 Oct 9;24(19):15005. doi: 10.3390/ijms241915005 (PMC10573222; doi:10.3390/ijms241915005)

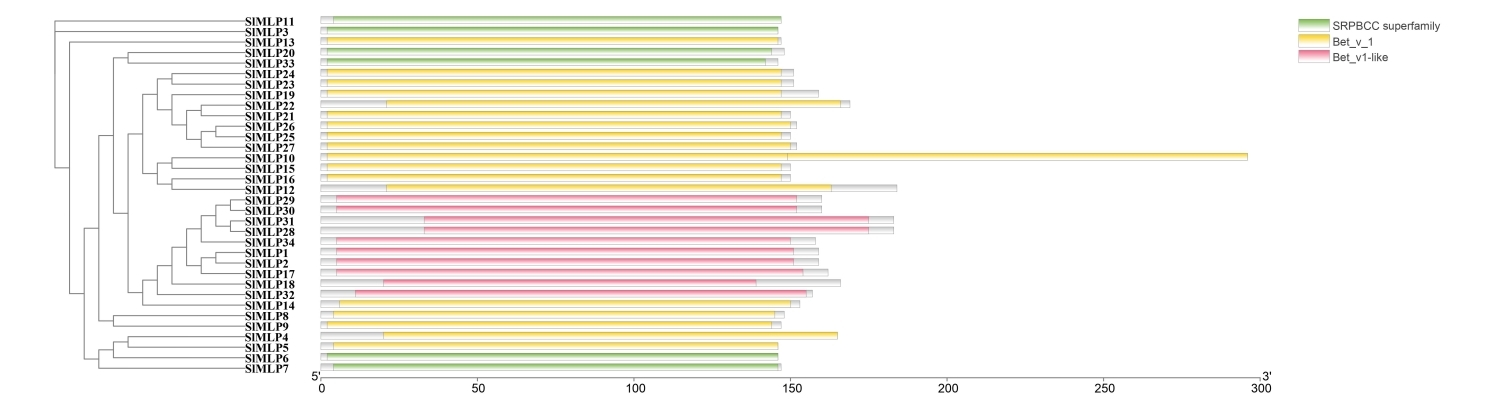

Supplement: Supplementary file 1 [file ijms-24-15005-s001.zip › Figure S1.tiff]

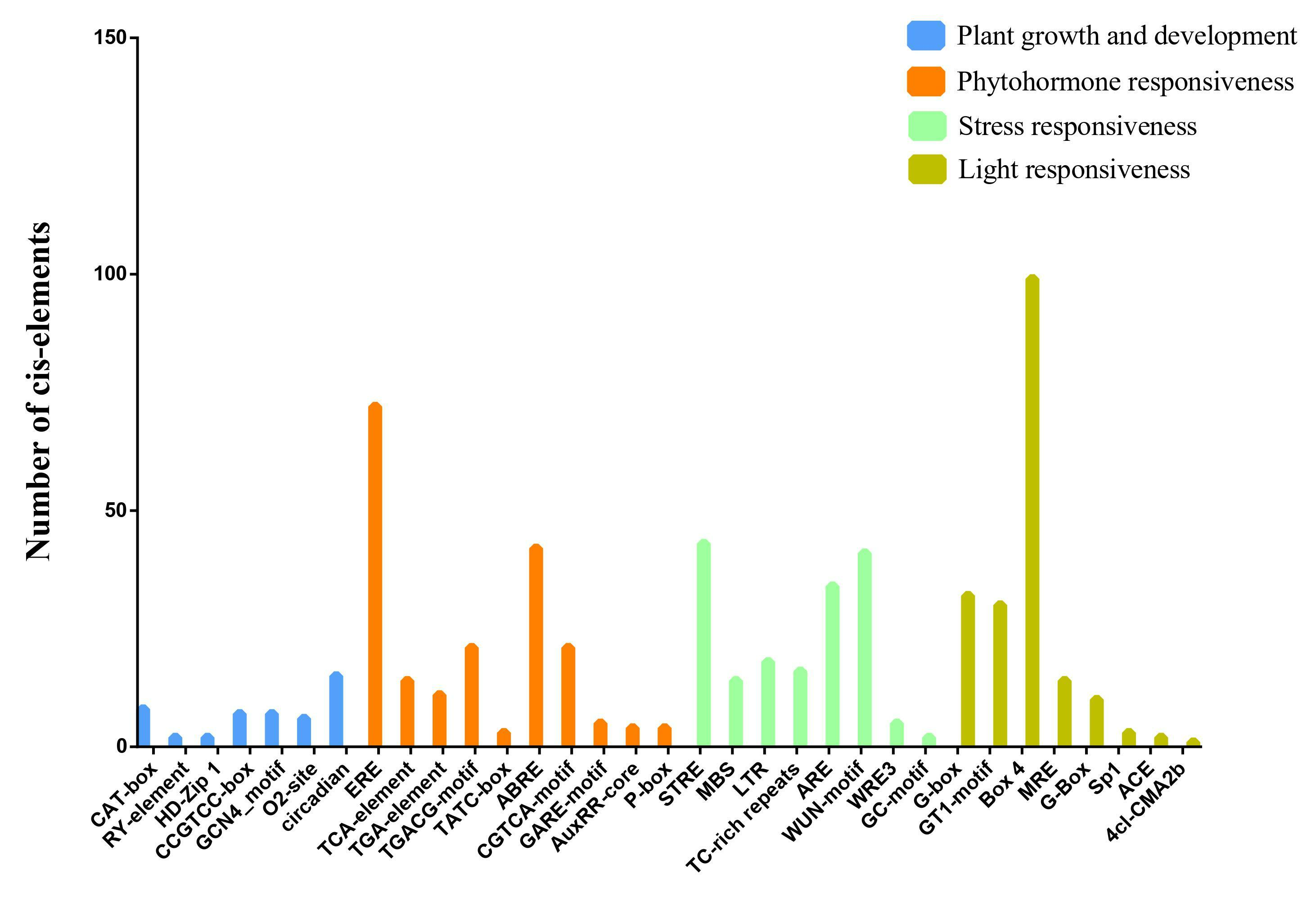

Supplement: Supplementary file 1 [file ijms-24-15005-s001.zip › Figure S2.tiff]
